# Targeting Natural Killer Cell Reactivity by Employing Antibody to NKp46: Implications for Type 1 Diabetes

**DOI:** 10.1371/journal.pone.0118936

**Published:** 2015-02-26

**Authors:** Rami Yossef, Chamutal Gur, Avishai Shemesh, Ofer Guttman, Uzi Hadad, Shlomo Nedvetzki, Antonija Miletić, Karen Nalbandyan, Adelheid Cerwenka, Stipan Jonjic, Ofer Mandelboim, Angel Porgador

**Affiliations:** 1 The Shraga Segal Department of Microbiology, Immunology and Genetics, Faculty of Health Sciences, Ben-Gurion University of the Negev, Beer Sheva, Israel; 2 The National Institute for Biotechnology in the Negev, Ben-Gurion University of the Negev, Beer Sheva, Israel; 3 The Lautenberg Center for General and Tumor Immunology, Institute for Medical Research Israel-Canada, Hebrew University-Hadassah Medical School, Jerusalem 91120, Israel; 4 Department of Medicine, Hadassah-Hebrew University Hospital, Jerusalem 91120, Israel; 5 Department of Clinical Biochemistry and Pharmacology, Faculty of Health Sciences, Ben-Gurion University of the Negev, Beer Sheva, Israel; 6 BioLineRx Ltd., 19 Hartum Street, P.O. Box 45158. Jerusalem 91450, Israel; 7 Center for Proteomics and Department for Histology and Embryology, School of Medicine, University of Rijeka, 51000 Rijeka, Croatia; 8 Pathology Department, Soroka Medical Center, Beer Sheva, Israel; 9 Innate Immunity Group, German Cancer Research Center, Heidelberg 69120, Germany; University of Siena, ITALY

## Abstract

Natural killer (NK) cells belong to the innate lymphoid cells. Their cytotoxic activity is regulated by the delicate balance between activating and inhibitory signals. NKp46 is a member of the primary activating receptors of NK cells. We previously reported that the NKp46 receptor is involved in the development of type 1 diabetes (T1D). Subsequently, we hypothesized that blocking this receptor could prevent or hinder disease development. To address this goal, we developed monoclonal antibodies for murine NKp46. One mAb, named NCR1.15, recognizes the mouse homologue protein of NKp46, named Ncr1, and was able to down-regulate the surface expression of NKp46 on primary murine NK cells following antibody injection *in vivo*. Additionally, NCR1.15 treatments were able to down-regulate cytotoxic activity mediated by NKp46, but not by other NK receptors. To test our primary assumption, we examined T1D development in two models, non-obese diabetic mice and low-dose streptozotocin. Our results show a significantly lower incidence of diabetic mice in the NCR1.15-treated group compared to control groups. This study directly demonstrates the involvement of NKp46 in T1D development and suggests a novel treatment strategy for early insulitis.

## Introduction

Natural killer (NK) cells of the innate immune system provide primary immune defense against cancer and pathogens [1–4]. Their activity is controlled by a multitude of inhibitory and activating NK cell receptors [1]. Instigation of inhibitory receptors by major histocompatibility complex (MHC) class I molecules on healthy cells suppresses NK cell effector functions [5,6]. The inhibitory receptors recognize mainly MHC class I proteins but also additional molecules [5]. Activating receptors recognize various ligands that are tumor-derived, stress-induced, pathogen-derived, or self-ligands [7,8]. Several activating receptors are expressed by NK cells, among them NKG2D and natural cytotoxicity receptors (NCRs). The human NCR family includes three members named NKp30, NKp44 and NKp46 [1]. Mice express orthologous receptor only for the human NKp46 (Ncr1) [9].

We recently reported that the murine activating receptor NKp46 (NCR1) is essential for the development of type 1 diabetes (T1D) [10], an autoimmune disease prevalent throughout the Western world. Currently the common treatment of this disease is based on a daily insulin injection. It is well known that T1D development is T-cell dependent, yet several studies have shown cytolytic activity of NK cells against pancreatic islet beta cells (β-cells) and their involvement in the development of the disease [11–15]. In particular, we have shown the involvement of NKp46 in NK contribution to T1D. We demonstrated that both human and mouse β-cells express ligands for NKp46 in either healthy or T1D patients [10,16]. We showed that development of experimental T1D was impaired in *Ncr1* ^*gfp/gfp*^ mice that lack the expression of mNKp46. Additionally, we showed that employing active immunization to block NKp46, through immunizing with recombinant NKp46, inhibited the development of T1D in murine models [10]. These findings motivated us to explore new therapeutic approaches for T1D based on manipulation of NKp46 function.

In order to accomplish this, one tactic would be to block the NKp46 ligands. However, the precise nature of NKp46 ligands is not fully revealed. Several reports have shown that NKp46 recognizes cellular ligands expressed on tumor cells, dendritic cells, viral-infected cells, and Langerhans β-cells [17,18]. Currently identified NKp46 ligands include viral hemagglutinins [19], the cellular ligand vimentin [20] and the cellular co-ligands heparan sulfate proteoglycans (HSPG) [21–23]. However, these two cellular ligands hardly present a suitable target for manipulation of NKp46 function through blocking of target cellular ligand.

Therefore, in the current study we investigated the methodology of antibody-mediated manipulation of the NKp46 function. We developed and characterized a new anti-murine NKp46 mAb named NCR1.15. Treatment of mice with NCR1.15 did not deplete NK cells, but suppressed their NKp46-mediated function. In accordance, NCR1.15 treatment of T1D-prone mice significantly prolonged the time to T1D development.

## Research Design and Methods

### Cells

Cells that were used in this study: YAC-1, murine lymphoma (TIB-160, ATCC); PD1.6, murine thymic virus-induced lymphoma [24]; Ba/F3-Rae1ε mouse pro-B lymphocyte ectopically expressing Rae-1ε NKG2D ligand [25]; BW-hNKp46 and BW-mNKp46 T-cell lymphoma ectopically expressing the murine or the human NKp46 [19].

### Production of NCR1.15 mAb

*Ncr1* ^*gfp/gfp*^ 129/sv/J mice, lacking the expression of endogenous mNKp46, were used for the production of mouse monoclonal antibodies against mNKp46. These mice were immunized twice with 100ug/mice of mNKp46-Ig fusion protein, followed by a boost immunization and a subsequent fusion with the mouse Sp2/0 cell line. First hybridoma screening for specific antibodies was performed using ELISA. Positive hybridomas were selected and cloned several times to ensure monoclonality. Antibodies obtained from these clones were further characterized using different techniques.

### Antibodies and Fusion proteins

The following antibodies were used in this study: BioLegend—anti-NKp46 mAb (clone 29A1.4), anti-CD3ε (clone145–2C11), anti-NK1.1 (clone PK136), anti-CD49b (clone DX5), anti-CD27 (clone LG.3A10), anti-NKG2D (clone C7), anti-CD107a/LAMP-1 (clone 1D4B); anti-CD11b (eBioscience, clone M1/70); anti-human NKp46 (461-G1,[26]); mouse IgG1, κ control for injections, rat IgG2a, κ isotype control for FACS (BioLegend, clone RTK2758). The Production of mNKp46-Ig, LIR1-Ig and mNKG2D-Ig as previously described [25,27,28].

### ELISA

To determine the specific binding between NCR1.15 and the mNKp46 receptor plates were coated overnight at 4°C with 5μg/ml of the recombinant proteins. Blocking buffer (PBS supplemented with 10%FBS) was applied for 2 hours at room temperature, after which plates were washed with PBS with 0.05% Tween 20 (PBST) and incubated with 2 μg/ml of NCR1.15, 461-G1or PBS for 2 hours at room temperature. Following washing with PBST biotin-conjugated sheep anti-mouse IgG (GE Healthcare, NA931V) was added for 1 hr at 1:750 dilution. Following washing streptavidin-HRP (Jackson, 016-030-084) diluted 1:1000 was added for 30 min. Following washing TMB (DAKO, S1599) was added optical density was read at 650 nm (Thermo Electron Corporation Multiskan Spectum).

### Bimolecular interaction analysis

A BIAcore 3000 device fitted with CM5 sensor chips (BIAcore, Uppsala, Sweden) was used for studying the interactions between NKp46 and NCR1.15 in conjunction with BIAevaluation software (v4.1). To activate the chip, we used the EDC/NHS amine coupling procedure according to the manufacturer’s protocol (BIAcore), followed by addition of NKp46, which was immobilized in the different flow cells, followed by blocking the free active groups with 1 M ethanolamine. Different analyte concentrations were injected, each followed by regeneration of the surface using 10 mM NaOH. Data were analyzed using a 1:1 Langmuir binding model.

### CD107a degranulation assay

Spleen lymphocytes were isolated 3 days following treatment and NK cells were purified using Mouse NK Cell Enrichment Kit (Stemcell Technologies, #19755). 5X10^5 purified NK cells were co-incubated with target cells for 2–4 hours in the presence of 0.1μg APC-coupled anti-CD107a antibody; next cells were stained for CD3ε, NK1.1 and CD107a and analyzed using FACSCantoII (BD Biosciences).

### Quantitative Real Time PCR (qRT-PCR)

Total RNA was extracted from Fresh spleen tissue using the RNeasy Mini Kit (cat# 74104, Qiagen Ltd). RNA samples were stored at-80°C until further use. RNA concentration and purity was assessed by spectrophotometry (NanoDrop ND1000 v3.7.1; NanoDrop Technologies, Delaware, USA). cDNA synthesis was performed using the High Capacity cDNA Reverse Transcription Kit (cat# 4368814, Applied Biosystems. cDNA samples were stored at-20°C until further use were. qRT-PCR reactions were performed using the Power SYBR Green PCR Master Mix (cat# 4367659, Applied Biosystems) and experiments were performed using the Applied Biosystems 7500 Real Time PCR system, software v2.0.5. Reactions cycling protocol: 95°C for 10 min, then 40 cycles with a 2-step program (95°C for 15s, 60°C for 1 min). Expression levels of *ncr1* (target gene) were normalized to *β actin* (reference gene). *ncr1* Fw: 5- CTAGGGCTCACAGAGGGACATAC-3, *ncr1* Rev 5- CAACACCTCCTGTGATGAGTAGT-3, *β actin* Fw: 5-GCATTGTTACCAACTGGGAC-3, *β actin* Rev: 5-GGTCTCAAACATGATCTGGG-3.

### Animals and NOD model

6–8 weeks old C57BL/6 female mice were purchased from Harlan Laboratories (Rehovot, Israel). For the T1D model in non-obese diabetic (NOD) strain 6–7 weeks old female mice were injected intraperitoneally (i.p.) with 100μg antibody per mouse every other day for 16 weeks and glucose levels were monitored. *Ncr1* ^*gfp/+*^ heterozygous mice expressing GFP under the control of *Ncr1* promoter in one allele [29] were used to assess the promoter activation levels. C57BL/6-Tg (UBC-GFP) mice were used for several CD107a de-granulation assays. Theses transgenic mice express enhanced Green Fluorescent Protein (EGFP) under the direction of the human ubiquitin C promoter (UBC) in all tissues (Jackson laboratory). All mice used in this study were bred and maintained in the local animal care facility, approved by the Institutional Animal Care and Use Committee (IACUC) of Ben-Gurion University of the Negev (BGU). All experimental procedures were reviewed and approved by the Institutional Animal Care and Use Committee (IACUC) of Ben-Gurion University of the Negev (BGU’s IACUC) according to specified protocols that aim to ensure animal welfare and reduce suffering (permit: IL-58–09–02).

### Low-dose streptozotocin (LDSTZ)-induced diabetes

Sex- and age-matched, 9 weeks mice were injected i.p. for 5 consecutive days with streptozotocin (STZ) (Sigma, S0130) dissolved in citrate buffer, pH 4.5, at a concentration of 50 mg · kg^-1^ body weight. Day zero was defined as the first day of STZ treatment. 50μg of NCR1.15 were inoculated i.p. at days-2 and 5. Glucose concentrations were measured using glucometer (Abbott Diabetes Care, FreeStyle Lite) every other day. Statistical analysis was done by log-rank Kaplan-Meier analysis.

### Isolation of primary β-cells

Pancreata were inflated with 1 mg/ml collagenase type XI (Sigma), excised, and incubated for 27–35 min at 37°C. Digested pancreata were vortexed and filtered through a 500-μm sieve and the pellet washed in HBSS containing 0.5% BSA (Sigma). The pellet was re-suspended in RPMI medium 1640 (Gibco, 21875–034). Islets were collected on a 100-μm cell strainer (BD, Falcon) and individual islets were picked by hand under the microscope and further dispersed into single cells [30] Islets’ single cell preparations were first pooled and only then divided among the different treatment groups.

## Results

### NCR1.15 mAb specifically recognizes mouse NKp46

**Fig 1:**
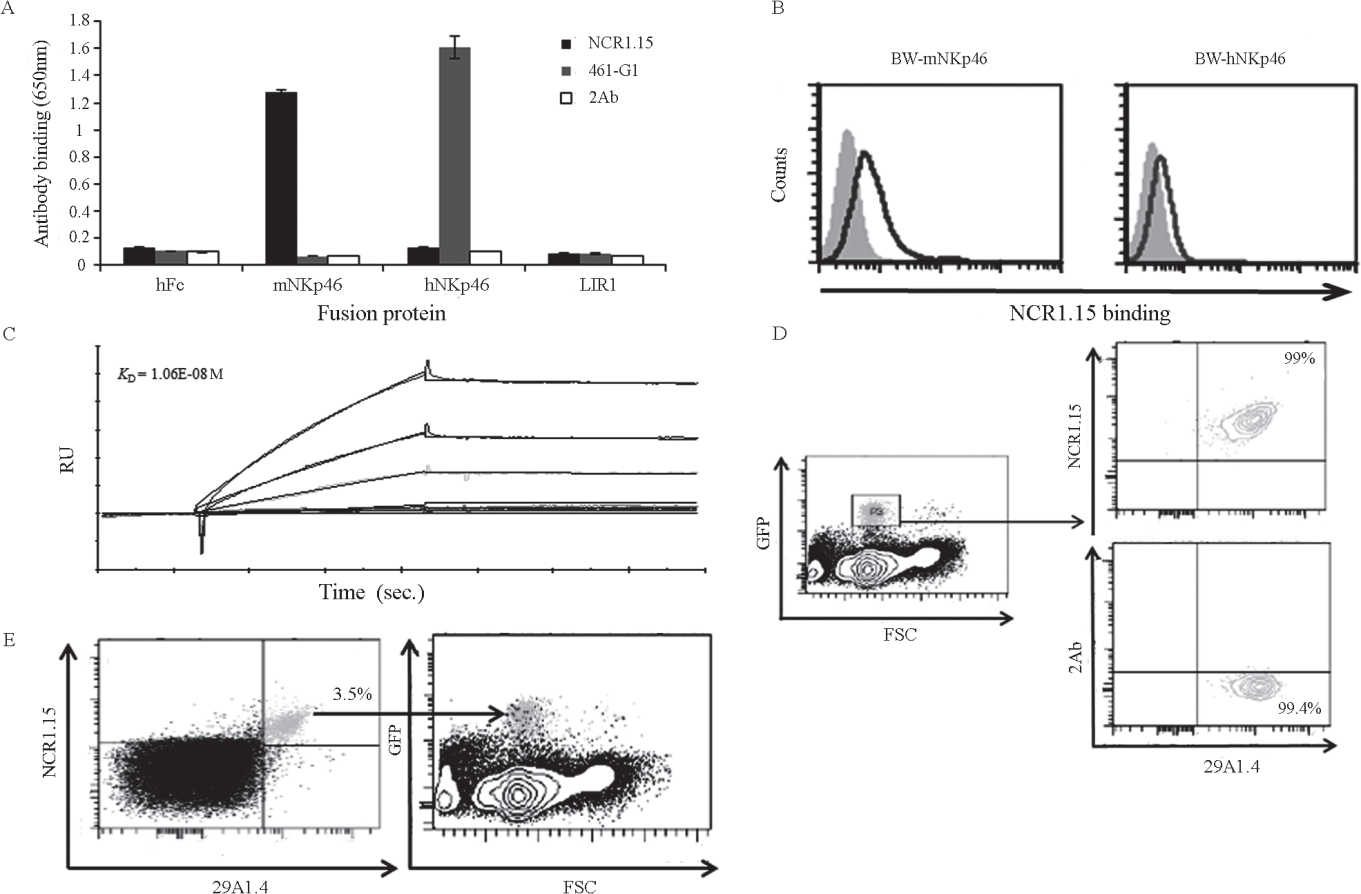
Specific binding of NCR1.15 monoclonal antibody to murine NKp46. (A) ELISA plates were coated with different NK-fusion receptors followed by incubation with NCR1.15 or 46–1G1 mouse antibodies, the binding was detected with anti-mouse IgG HRP. Data from one representative of three independent experiments are shown. (B) FACS analysis of NCR1.15 binding to BW cells expressing mNKp46 or hNKp46. (C) Kinetics of NCR1.15 binding to mNKp46-Ig; the results from the Biacore analysis are expressed in resonance units (RU) against time. Dose-dependent NCR1.15 were injected over the immobilized fusion Ig’s surface at increasing concentrations ranging from 0 to 250nM. (D-E) FACS analysis of *NCR1* ^*gfp/+*^ splenocytes stained for NKp46 with 29A1.4 and NCR1.15 clones. Data from one representative of three independent experiments are shown. Bars, ±SD.

To test whether blocking of NKp46 (Ncr1 in mice) will attenuate T1D development, we developed an anti-mouse NKp46/Ncr1 IgG1,κ mAb (termed NCR1.15) that specifically recognizes recombinant mouse NKp46 (mNKp46) but not human NKp46 (hNKp46). As a control, we employed 461-G1 mAb that specifically recognizes hNKp46 but not mNKp46 [26] (Fig. 1A). Both mAbs did not bind to other recombinant NK receptors such as LIR1 or to the hFc which is fused to the recombinant NK receptors employed in the binding assays (Fig. 1A and data not shown). NCR1.15 also recognizes BW cells that were transfected to express mNKp46 compared to BW cells expressing hNKp46 (Fig. 1B). NCR1.15 displayed a characteristic binding curve to mNKp46 (*K*_*D*_ = 1.06E-08 M), indicating binding with moderate affinity (Fig. 1C). To test whether NCR1.15 specifically recognizes endogenous mNKp46 expressed by murine primary NK cells, we stained splenocytes from the functionally competent *Ncr1* ^*gfp/+*^ heterozygous mice expressing one allele encoding for mNKp46 and the other allele encoding for GFP under the control of *Ncr1* promoter in the other allele [29]. GFP-positive splenocytes were nearly all (99%) stained positively with NCR1.15 or with the commercial anti-mNKp46 mAb 29A1.4 and (Fig. 1D). Accordingly, the overwhelming majority of NCR1.15^+^ or 29A1.4^+^ single or double positives were also GFP positive (Fig. 1E). To summarize, NCR1.15 specifically stained mNKp46 expressed by murine NK cells.

### Single dose treatment with NCR1.15 reduces membrane-associated NKp46 expression and NKp46-mediated NK function

**Fig 2:**
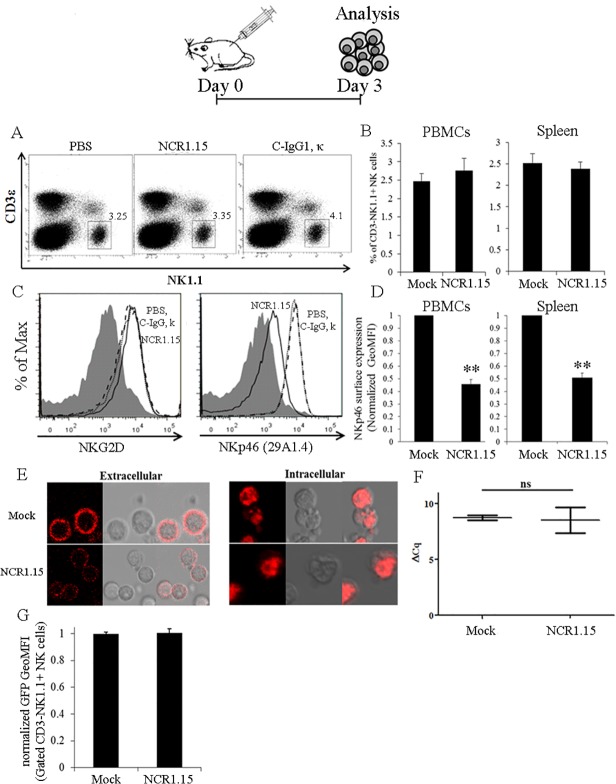
Single dose treatment with NCR1.15 down-regulates the surface expression of NKp46 on NK cells, but not the NKp46 transcript or spleen/blood NK levels. 3 days following i.p. injection of NCR1.15, isotype control or PBS splenocytes (A, B) and PBMCs (B) were stained and analyzed for CD3^-^NK1.1^+^ NK cells using flow cytometry. Data from one representative of five independent experiments are shown. The membrane associated NKp46 (C, D) and NKG2D (C) on NK cells were stained and analyzed using flow cytometry. (E) Representative confocal images of purified splenic NK cells stained for extra- and intracellular expression of the NKp46. (F) qRT-PCR of NKp46 transcripts in splenocytes harvested from treated mice. (G) GFP intensity (GeoMFI) expressed by NK cells from *NCR1* ^*gfp/+*^ following the various treatments. Data from one representative of three independent experiments are shown. ** *p*<0.01 by Student's unpaired *t*-test. Bars, ±SD.

To investigate the effect of anti-mNKp46 treatment *in vivo*, we first inoculated C57BL/6 mice i.p. with 100μg NCR1.15/mouse (single dose). Three days post inoculation, a fraction of NK cells, and NKp46 expression levels were monitored for splenocytes and PBMCs (Fig. 2). Fig. 2A shows a representative NK staining of splenocytes and Fig. 2B summarizes the NK staining results (CD3^-^NK1.1^+^) for splenocytes and PBMCs from four independent experiments. The NK fraction in both the spleen and blood did not change significantly following a single dose injection of NCR1.15 compared to mock injection (isotype control or PBS). In parallel, we tested membrane-associated NKp46 expression following single dose injection of NCR1.15. Fig. 2C shows a representative NKp46 and NKG2D staining for CD3^-^NK1.1^+^ NK cells purified from splenocytes that were harvested 3 days after inoculation. Note that NKp46 staining for the experiments described in Fig. 2 was performed with commercial 29A1.4 mAb that its binding did not overlap with NCR1.15 (Fig. 1D, E). NKp46 cell surface levels, but not NKG2D levels, were considerably reduced following NCR1.15 injection as compared to PBS or isotype control injections (Fig. 2C). Fig. 2D summarizes the NKp46 staining results for CD3^-^NK1.1^+^NK-gated splenocytes and PBMCs from four independent experiments. Cell-membrane levels of NKp46 were significantly down-regulated following single dose injection of NCR1.15 compared to mock injection (near 2-fold change). However, we also analyzed NKp46 expression on CD3^+^NK1.1^+^ NKT cells, knowing that the NKp46 is expressed on subset of these cells [31], and got similar reduction levels of NKp46 expression with no change in NKT levels (data not shown). Further, to investigate whether this down-regulation at the cell surface reflects suppression of NKp46 production or internalization of the protein, we studied NKp46 localization employing confocal microscopy. Intracellular expression of NKp46 was similar in the NK from either NCR1.15- or mock-treated mice, while membrane-associated staining was reduced in the NK from NCR1.15-treated mice (Fig. 2E). Thus, it is probable that NCR1.15 treatment neither suppressed NKp46 protein synthesis nor enhanced NKp46 internalization.

Following the aforementioned results in the protein level, we hypothesized that single injection of anti-NKp46 would not suppress the activity of the NKp46 promoter or induce degradation of NKp46-encoding RNA. Indeed, qPCR analysis of NKp46-encoding mRNA showed that NCR1.15 treatment did not affect the levels of transcribed NKp46 (Fig. 2F). To better address the question for the NKp46 promoter activity, we employed *Ncr1* ^*gfp/+*^ heterozygous mice expressing one allele encoding for mNKp46 and the other allele encoding for GFP under the control of *Ncr1* promoter. NCR1.15 treatment did not alter GFP levels in NK cells compared to mock-treated mice (Fig. 2G), indicating that a single dose injection of anti-NKp46 did not affect *Ncr1* promoter activity. To summarize, single dose injection of NCR1.15 significantly reduced membrane-associated levels of NK-expressed NKp46 but did not affect NKp46 transcription or translation.

**Fig 3:**
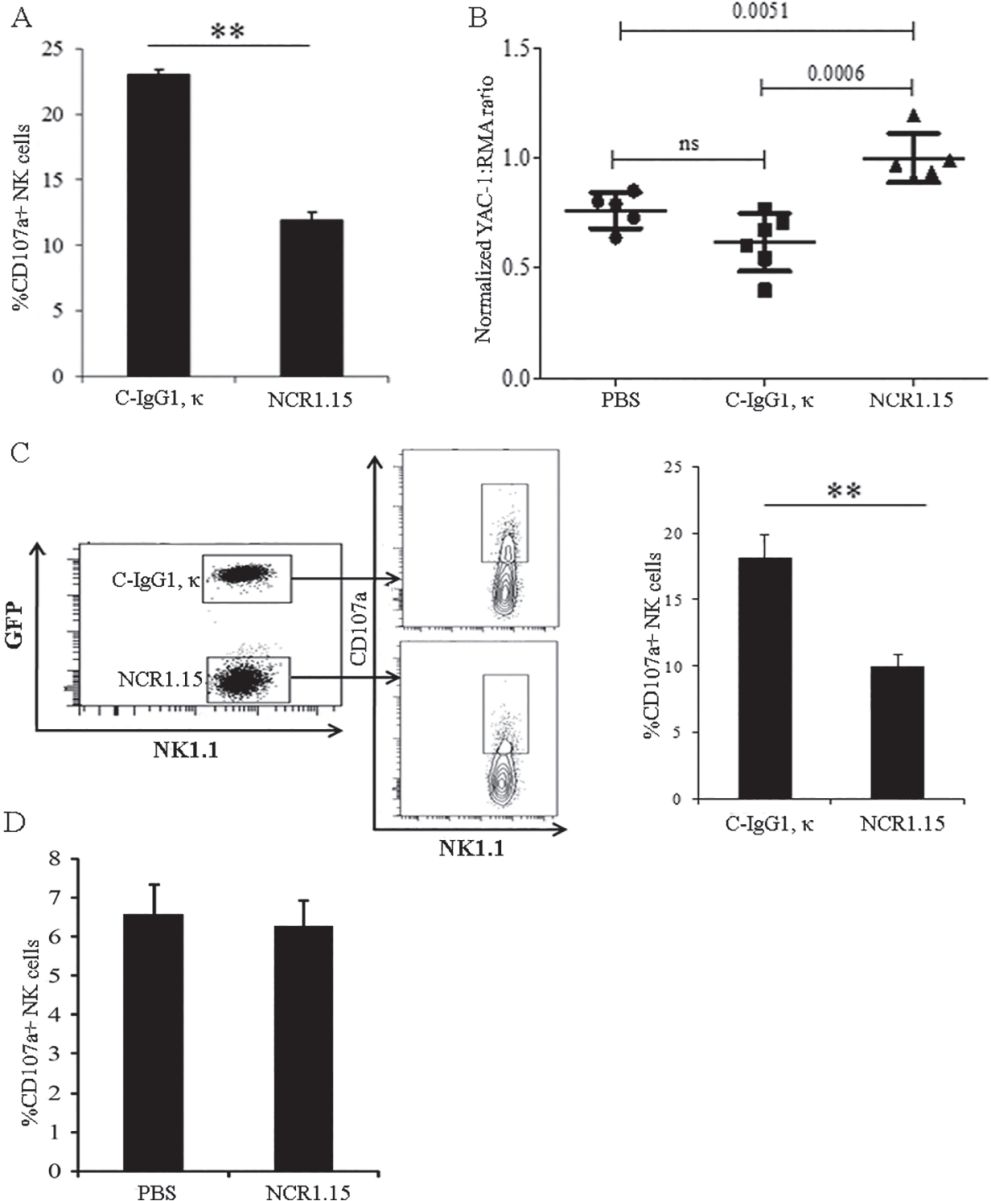
Single dose treatment with NCR1.15 inhibits NKp46-mediated activity on NK cells. (A) 3 days following i.p. injection of NCR1.15, isotype control or PBS purified splenic NK cells were co-incubated for 2–4 hours with YAC-1 target cells in the presence of anti-CD107a antibody. CD107a surface levels on NK cells were assessed by FACS. (B) Labeled YAC-1 and RMA cells were injected into the tail vein of 5 NCR1.15-, c-IgG1 κ- or PBS-treated mice. Cells in the lungs were quantified 3–4 hours following the injection using FACS analysis. Data from one of two independent experiments are shown. (C) 3 days following mice i.p. injection c-IgG1 κ-treated *UBC-GFP* and NCR1.15-treated C57BL/6 WT purified splenic NK cells were co-incubated with PD1.6 target cells in 1:1:1 ratio (E_1_:E_2_:T) with the presence of anti-CD107a antibody for 3–4 hours. CD107a surface levels on NK cells were assessed by FACS. (D) Purified splenic NK cells harvested from control- or NCR1.15-treated mice 3 days after the treatment were co-incubated with Ba/F3-Rae1ε target cells in 1:1 E:T ratio for 3–4 hours. CD107a surface expression levels were analyzed using FACS. Data from one representative of three independent experiments are shown. ** *p*<0.01 by Student's unpaired *t*-test. Bars, ±SD.

We then studied whether down-regulation of membrane-associated NKp46 affects NK activity using YAC-1 target cells, which are sensitive to NK activity. CD3^-^NK1.1^+^NK cells purified from splenocytes harvested 3 days after NCR1.15 inoculation significantly reduced vesicle degranulation following incubation with YAC-1 target cells (Fig. 3A). To test whether this observation is correct *in vivo*,we mixed two fluorescently labeled target cells, NK-sensitive YAC-1 and NK insensitive RMA cells (1:1 ratio), and injected them intravenously 3 days after NCR1.15/mock treatment of mice. YAC-1 and RMA cells were labeled with different fluorescent probes; thus we could differ between the two targets in the same lung. Lung clearance assay of YAC-1 cells normalized on RMA cells was significantly lower in NCR1.15-treated mice compared to PBS- or isotype control-treated mice (Fig. 3B). We extended our studies to PD1.6 target cells that manifest sensitivity to NKp46-mediated NK function [32]. To better compare effector cells, we mixed PD1.6 target cells with effector NK cells purified from both isotype control- and NCR1.15-treated mice (1:1:1 ratio, E1:E2:T) and tested NK cell degranulation. One effector NK population was derived from WT C57BL/6 mice and the other was derived from *UBC-GFP* C57BL/6 mice. Therefore, by detecting GFP, we could differ between the two NK populations using a flow cytometry-based degranulation assay in the same well. Fig. 3C shows a representative assay in which *UBC-GFP* mice were mock-treated, and WT mice were NCR1.15-treated. With no target cells, the degranulation level was near zero and similar between the two mixed NK effector populations. Following incubation with target PD1.6 cells, the degranulation of mock-treated NK cells was significantly higher compared to the NCR1.15-treated NK cells in the mixed population (Fig. 3C).

To further investigate whether this reduction in NK activity is general or NKp46-associated, we employed as target cells Ba/F3 cells that express null levels of ligands to NKp46 and NKG2D, transfected with the NKG2D ligand Rae1ε (Ba/F3-Rae1ε, Fig. 3D). Therefore, we could test whether the suppressing effect of NCR1.15 treatment is restricted to NKp46-mediated NK function. With no target cells, the degranulation level was near zero and similar between the two NK effector populations. Following incubation with Ba/F3-Rae1ε target cells, degranulation was observed, but with no significant difference between NK cells derived from either mock- or NCR1.15-injected mice (Fig. 3D). To summarize, one injection of NCR1.15 specifically induced NKp46-associated reduction of NK function when tested 3 days after treatment.

### Treatment with NCR1.15 inhibited LDSTZ-induced diabetes

**Fig 4:**
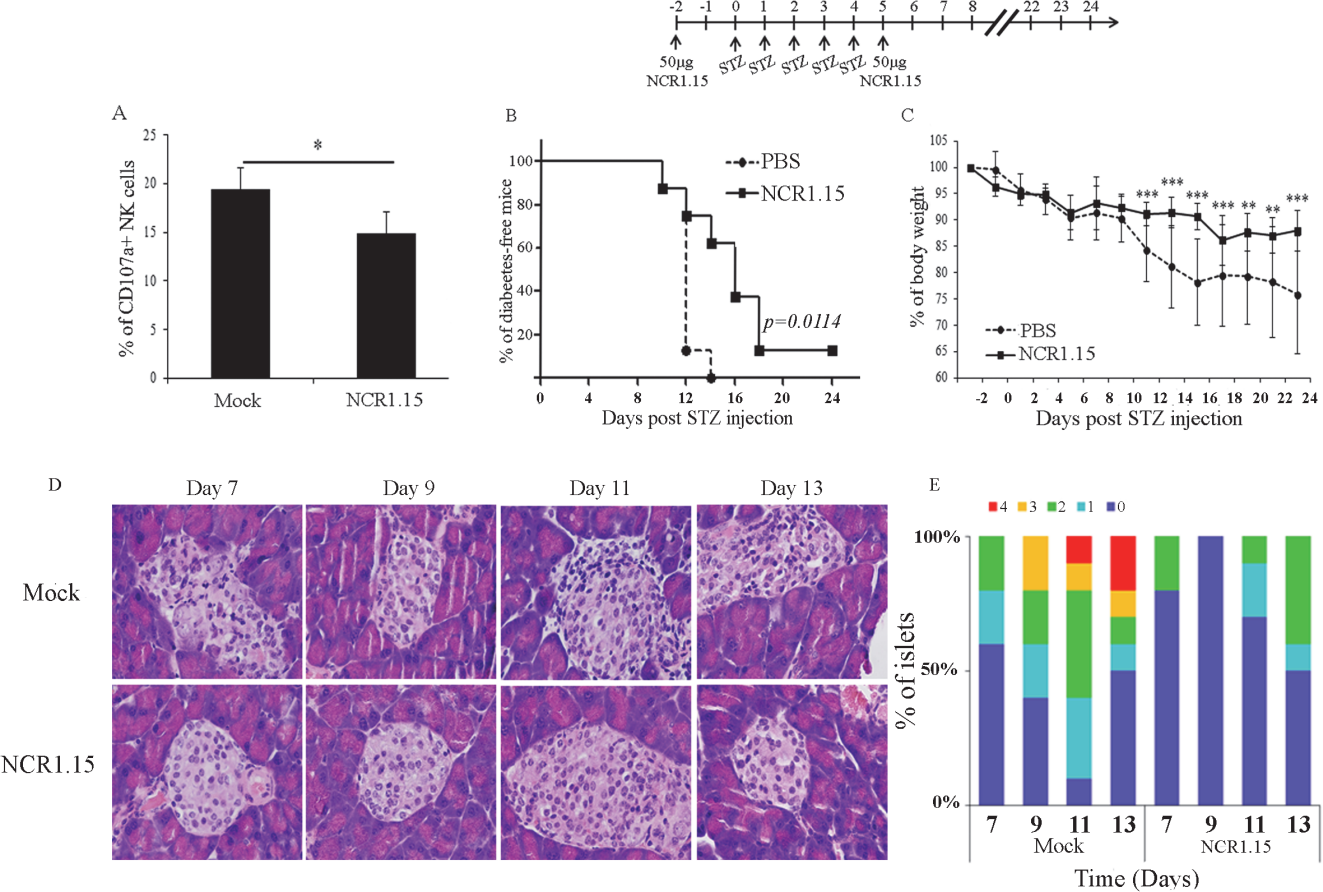
Short term treatment with NCR1.15 down-regulates NKp46-mediated activity on pancreatic β-cells and inhibits LD-STZ induced diabetes. (A) Purified splenic NK cells from treated cells were co-incubated for 2–4 hours with purified C57BL/6 β-cells in the presence of anti-CD107a antibody. CD107a surface levels on NK cells were assessed by FACS. * *p*<0.05 by Student's unpaired *t*-test. (B) LD-STZ induced diabetes development in C57BL/6 mice following 50μg injections at days-2 and 5. (*n* = 8). Animals were considered diabetic when blood glucose was ≥300mg/dl. *** *p*<0.005 Kaplan-M log-rank (Mental-Cox). (C) Body weight of mice in the indicated groups following the LD-STZ administration; body weight baselines at day 0 of the control and NCR1.15 groups were 19.2±0.86, 19.8±1 grams, respectively. * *p*<0.05, ** *p*<0.01, *** *p*<0.005 by Student's unpaired *t*-test. Bars, ±SD. (D) Representative H&E sections of the pancreas from sex&age-matched C57BL/6 mice treated with NCR1.15 or mock-treated (IgG1, κ) in LD-STZ induced diabetes model (magnification X40). (E) Summary of insulitis score over the days calculated as described in [37]. Score is quantified from 0 (low) to 4 (most severe).

We have shown that a single treatment with NCR1.15 was able to reduce NKp46-associated NK function on NKp46-sensitive target cell lines. Primary β-cells in the islets of Langerhans were reported to serve as targets to NK cells and that NKp46 is primarily involved [33]. Indeed, co-incubation of pancreatic primary beta cells from naïve mice with NK cells derived from NCR1.15 single injection treated mice, both from C57BL/6 origin, resulted in significantly reduced degranulation levels (Fig. 4A). We next investigated whether NCR1.15 short-term treatment could affect the early development of diabetes. An accepted model of experimental autoimmune diabetes in mice is the induction of diabetes by multiple injections of a low dose of streptozotocin (LDSTZ) [34–36]. A single NCR1.15 treatment comprises a single 100 μg/mouse injection. Here, we treated mice once with NCR1.15 (50 μg/mouse) followed by 5 consecutive days of i.p. injections with STZ and completed with additional NCR1.15 treatment (Fig. 4B, inset). The NCR1.15 treatment significantly impaired diabetes development. Hyperglycemia, defined as a non-fasting blood glucose concentration of ≥300 mg/dl in two sequential measurements, was significantly less severe in NCR1.15-treated mice compared to a mock treatment group (Fig. 4B). Accordingly, reduction in body weight of the NCR1.15-treated mice was significantly lower than that of the control mice, indicating the healthier status of NCR1.15-treated mice (Fig. 4C). Additionally, histological analysis of the pancreas showed reduced mononuclear infiltration and reduced insulitis score for the islets within NCR1.15-treated mice as compared to mock-treated group (Fig. 4D, E). To sum up, these results indicate that NKp46 is important for the development of diabetes and the destruction of islets in the LDSTZ model.

### Repeated long-term treatments with NCR1.15 inhibited T1D development in NOD mice

**Fig 5:**
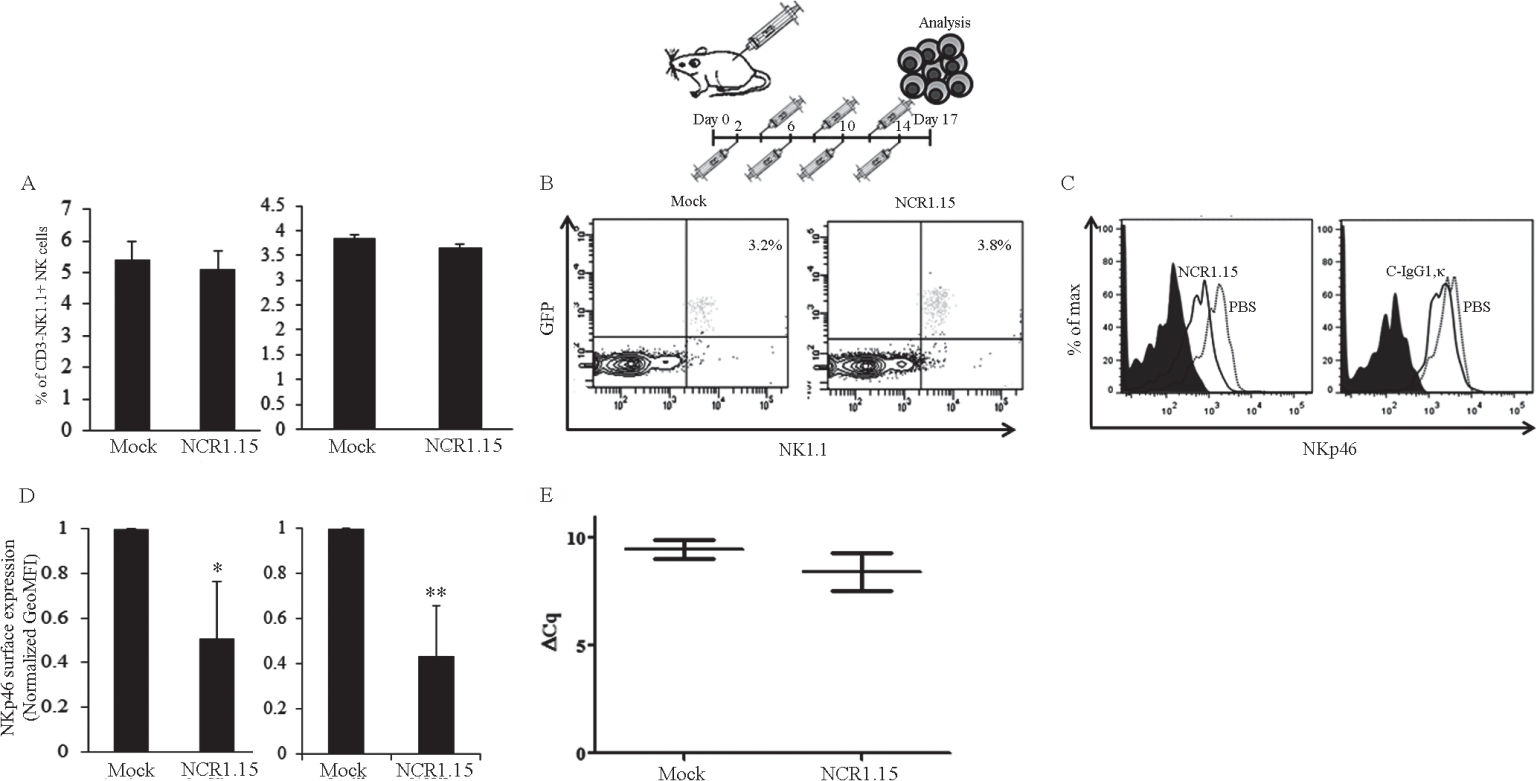
Prolonged multiple treatments with NCR1.15 down-regulates the surface expression of NKp46. Mice were injected i.p. 8 doses of PBS, 100 μg of NCR1.15 or cIgG1,κ every other day; 3 days after the last inoculation mice were sacrificed for further analysis. (A) C57BL/6 WT PBMCs and splenocytes were harvested and stained to detect the levels of CD3^-^NK1.1^+^ NK cells. Data from one representative of three independent experiments are shown. (B) PBMCs drained from *NCR1* ^*gfp/+*^ mice were analyzed for GFP expression following the prolonged repeated treatments. (C, D) Membrane associated NKp46 and NKG2D levels were analyzed on gated CD3ε^-^NK1.1^+^ NK cells. (E) *NCR1* transcript levels from treated mice splenocytes were assessed by qRT-PCR. Data from one representative of two independent experiments are shown. * *p*<0.05, ** *p*<0.01 by Student's unpaired *t*-test. Bars, ±SD.

We have previously shown that beta cells specifically express the ligand for the NK-activating receptor NKp46/Ncr1 and that NK cells kill beta cells in an NKp46/Ncr1-dependent manner [10,33]. Furthermore, we have shown that anti-mNKp46/Ncr1 antibodies generated through active immunization inhibit T1D development [10]. To recapitulate these observations, we investigated the effect of repeated NCR1.15 treatments in C57BL/6 mice. WT mice were treated with NCR1.15 every other day for two weeks and both PBMCs and splenocytes were tested 3 days following the last injection. Our results were very similar to those observed for one NCR1.15 inoculation (Figs. 2, 3). The fraction of NK cells in PBMCs and the spleen did not change between NCR1.15- and mock-treated mice (Fig. 5A). We also employed the protocol of prolonged multiple injections of NCR1.15 to *Ncr1* ^*gfp/+*^ mice and tested for the fraction of GFP-positive cells indicating NK fraction [20] and for GFP intensity indicating *Ncr1* gene promoter activity. Neither the numbers of GFP^+^ nor the GFP intensity was different between NCR1.15- and mock-treated mice (Fig. 5B). In contrast, membrane-associated NKp46 expression was significantly reduced in PBMCs and in splenocytes harvested from mice that had undergone prolonged multiple NCR1.15 treatments compared to mock-treated mice (Fig. 5C, D). As with the single NCR1.15 injection, this reduction was not due to reduced *Ncr1* gene promoter activity as deduced from qRT-PCR levels of the *Ncr1* transcript (Fig. 5E) and from the *Ncr1*-encoded GFP levels (Fig. 5B). Since treatment lasted for two weeks and mice were tested 3 days later, we could investigate whether NCR1.15 treatment conferred changes in NK subsets representing developmental NK stages. Antibodies to CD3, NK1.1, CD27, and CD11b were used to define CD3^-^NK1.1^+^CD11b^low^CD27^+^, CD3^-^NK1.1^+^CD11b^+^CD27^+^ and CD3^-^NK1.1^+^CD11b^+^CD27^low^ NK developmental stages. No significant difference was observed between NCR1.15- and mock- treatment for cell numbers among the NK developmental stages. In contrast, membrane-associated NKp46 levels were significantly reduced in NCR1.15-treated mice in all studied NK developmental stages as shown in S1 Fig.

**Fig 6:**
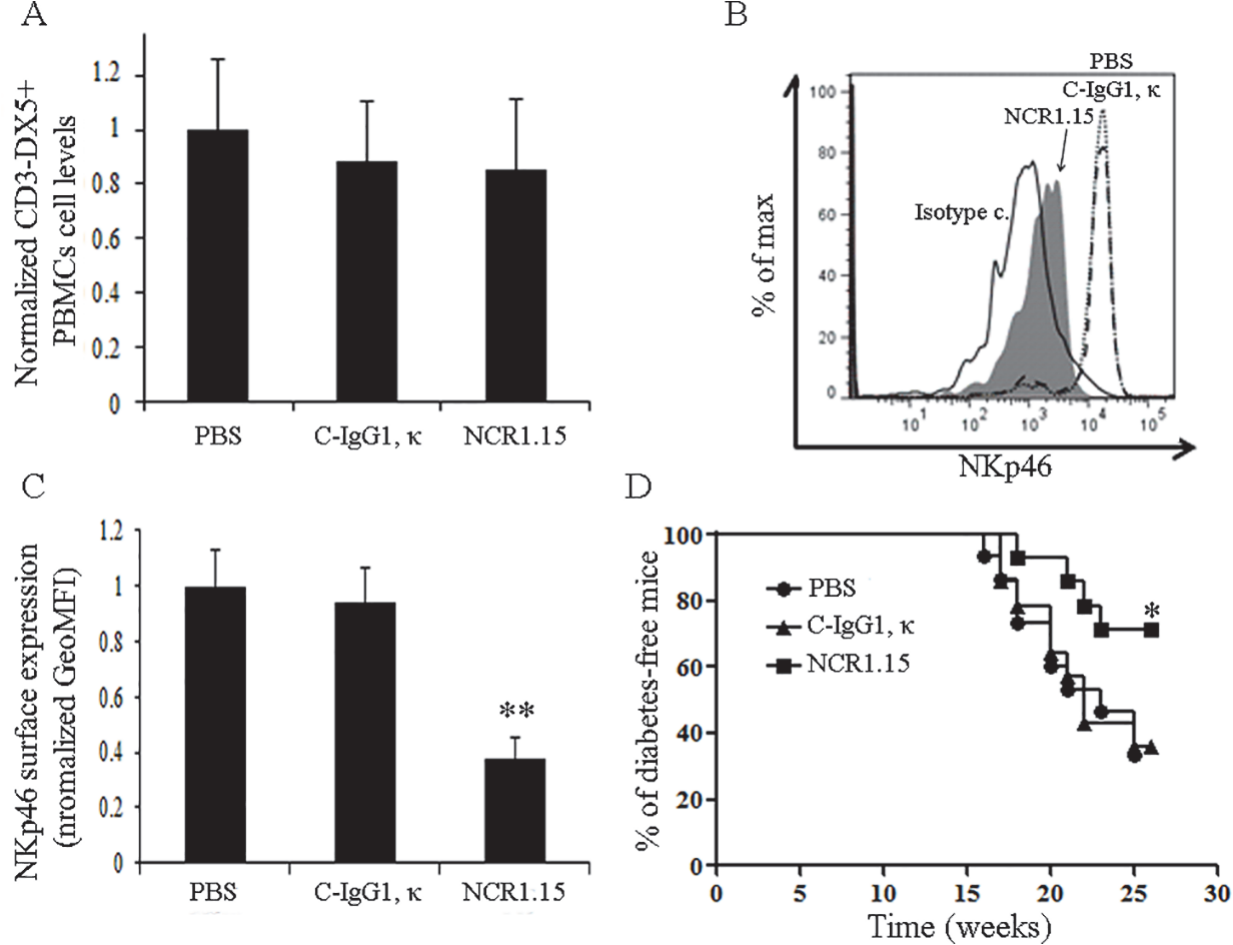
Treatment with NCR1.15 reduces NKp46-mediated NK activity on β-cells and inhibits T1D development. (A) 8 weeks after the first treatment NOD PBMCs drained from mice within the indicated groups were stained and analyzed for NK levels. NK cell levels were normalized to mock (PBS) treatment. (B) Representative histogram of membrane associated NKp46 levels on gated CD3ε-CD49b+ NK cells from the indicated groups. (C) Normalized GeoMFI of membrane associated NKp46 on gated NK cells, ** p<0.01 by Student's unpaired t-test. GeoMFI values were normalized to mock (PBS) treatment. (D) T1D development in NOD female mice in the indicated groups. (n = 14–15). Animals were considered diabetic when blood glucose was ≥250mg/dl. * p<0.05 Kaplan-M log-rank (Mental-Cox). Bars, ±SD.

We then studied repeated long-term NCR1.15 treatments in NOD mice. As for C57BL/6 mice, NCR1.15 treatment of NOD mice did not affect number of NK cells (Fig. 6A), but it did significantly reduce membrane-associated NKp46 expression (Fig. 6B, C). To directly demonstrate that blocking of NKp46 in NOD mice will block diabetes development, we injected NCR1.15 into NOD mice every other day (100μg per injection) beginning in week eight. As shown in Fig. 6D, repeated NCR1.15 treatment significantly inhibited T1D development in NOD female mice compared to treatment with PBS or isotype Ab control.

## Discussion

We have recently reported that NKp46, a primary activating receptor expressed by NK cells, recognizes ligands expressed by islet β-cells, and that in the absence of NKp46 diabetes development is inhibited [10,33]. In the current study, we investigated whether treatment of mice with NKp46 specific monoclonal antibody could down-regulate NKp46-mediated NK function and inhibit T1D development. We developed the NCR1.15 mAb, which specifically recognizes mNKp46 (Fig. 1) and showed that treatment of mice with NCR1.15 reduces NKp46 membrane-associated expression by NK cells (Fig. 2). This reduction was paralleled by reduced NKp46-mediated NK function (Fig. 3). NCR1.15 treatment did not reduce the fraction of NK cells in the blood or the spleen; neither did it affect the NKG2D-mediated function of NK cells (Figs. 2, 3). This NKp46-specific reduction in NK function was shown both *in vitro* and *in vivo* (Fig. 3). Finally, we showed that multiple treatments with NCR1.15 inhibited development of diabetes in the LDSTZ model and in NOD mice (Figs. 4, 6). Acquired histopathological data for the LDSTZ model indicate that development of insulitis is attenuated in NCR1.15-treated mice as compared to mock-treated mice (Fig. 4).

Targeted immunotherapy involving treatment with antibodies to activate immune cellular receptors and immune molecules holds great promise for the treatment and cure of autoimmune and inflammatory diseases [38,39]. In particular, Ab-mediated modulation of immune effector receptors and receptor-expressing cells have been widely studied for the TCR [38,40].Modulation of the TCR-CD3ζ by specific antibodies has been suggested to down-regulate T cell function through (i) antigenic modulation (suppression) of the cell-surface TCR, (ii) depletion of the T cells, and /or (iii) the anergy of T cells [40,41]. We have shown that treatment with anti-NKp46 resulted in antigenic modulation of NK-expressed NKp46, but not in general NK depletion. Anergy wise, our treatment induced down-regulation of NKp46-specific NK function, but it did not induce general NK anergy, as tested by NKG2D function (Fig. 3D). Down-regulation of NK function following exposure to anti-NKp46 mAb has also been reported by Jewett et al. [42]. However, Narni-Mancinelli *et al*. reported a more complex phenotype [43]: short time treatment with anti-NKp46 resulted in reduced NKp46-mediated NK function in accordance with our results and other reports [42] even though long time-treatment caused enhanced NKp46-mediated NK function [43]. This observation for long-time treatment was not reported by others nor us, and could stem from the diphtheria toxin-mediated whole NK depletion performed before the long time treatment [43]. Yet entire NK cell depletion before treatment with anti-NKp46 does not represent a plausible treatment regimen in humans.

Several groups reported that treatment of T1D with mAb specific to CD3 resulted in a significant therapeutic effect through the antigenic modulation of the TCR-CD3ζ complex (reviewed in [40]). However, others have shown that this therapeutic effect was due to induction of T cell anergy or apoptosis of activated T cells [44]. T1D disease is considered to be mainly driven by the activation of T-lymphocytes against pancreatic β-cells [45]. However, findings support the view that T1D can be regarded as an innate inflammatory disease affecting the entire pancreas, but with its main clinical manifestations emanating from the loss of the insulin-producing cells [46]. In this respect, we, among others, have shown that NK cells contribute to the early stage of T1D development [10,20]. The question then arose whether treatment with mAb targeting NK activating receptors can have a therapeutic benefit for T1D. The NKG2D receptor is a primary activating receptor for NK cells and a co-stimulatory receptor for T cells. Blockage of NKG2D prevented T1D in NOD mice. The mechanism reported involved blockage of NKG2D receptors to their ligands, as well as internalization of the receptor. However, no depletion of NK cells or CD8^+^ T cells was observed [47]. NKp46 is a major NK activating receptor, and it is mostly restricted to NK cells compared to the broader expression spectrum of NKG2D. We demonstrated that β-cells both human and mouse islets express ligand(s) for NKp46 [10,33]. We also showed that in the absence of NKp46 diabetes development is inhibited. We then proved that NKp46 is involved in the direct killing of human islets β-cells that are intended to be used for transplantation [10,33]. The NKp46 ligand is detected on healthy mouse β-cells, but we demonstrated that NK cells were absent from the vicinity of the islets in healthy mice and were detected in situ in proximity with β-cells in NOD mice [10,33]. In this study, we have investigated a treatment based on the inoculation of mAb specific for NKp46. Following treatment, NK cells were not depleted nor did they undergo general anergy. Even so, we observed a very clear antigenic modulation of membrane-associated NKp46 and NKp46 specific NK function that was followed by a significant impairment of T1D development. It will be interesting to further explore whether the combination of both anti-NKp46 and anti-NKG2D mAbs will lead to an even more pronounced delayed onset of this disease.

To summarize, in this study, we have shown that anti-NKp46 treatment was sufficient to significantly delay diabetes early development in LDSTZ and NOD models. Both, short-term and repeated long-term treatments with anti-NKp46 mAb resulted in an NKp46-specific impairment of NK function without NK depletion. This treatment represents a pinpoint-targeted therapeutic approach intended to specifically remove/reduce the NKp46 component in NK function neither harming non-NKp46 mediated NK activity nor affecting T cells.

## Supporting Information

S1 Fig Membrane-associated NKp46 levels in different NK developmental stages (Y-axis in histogram representation). The membrane-associated NKp46 expression was significantly reduced in NCR1.15 treated mice comparing to control treatments in all studied NK developmental stages.
(TIF)
