# Supplementary figures and images for: Targeting Natural Killer Cell Reactivity by Employing Antibody to NKp46: Implications for Type 1 Diabetes
Source: PLoS One. 2015 Feb 26;10(2):e0118936. doi: 10.1371/journal.pone.0118936 (PMC4342013; doi:10.1371/journal.pone.0118936)

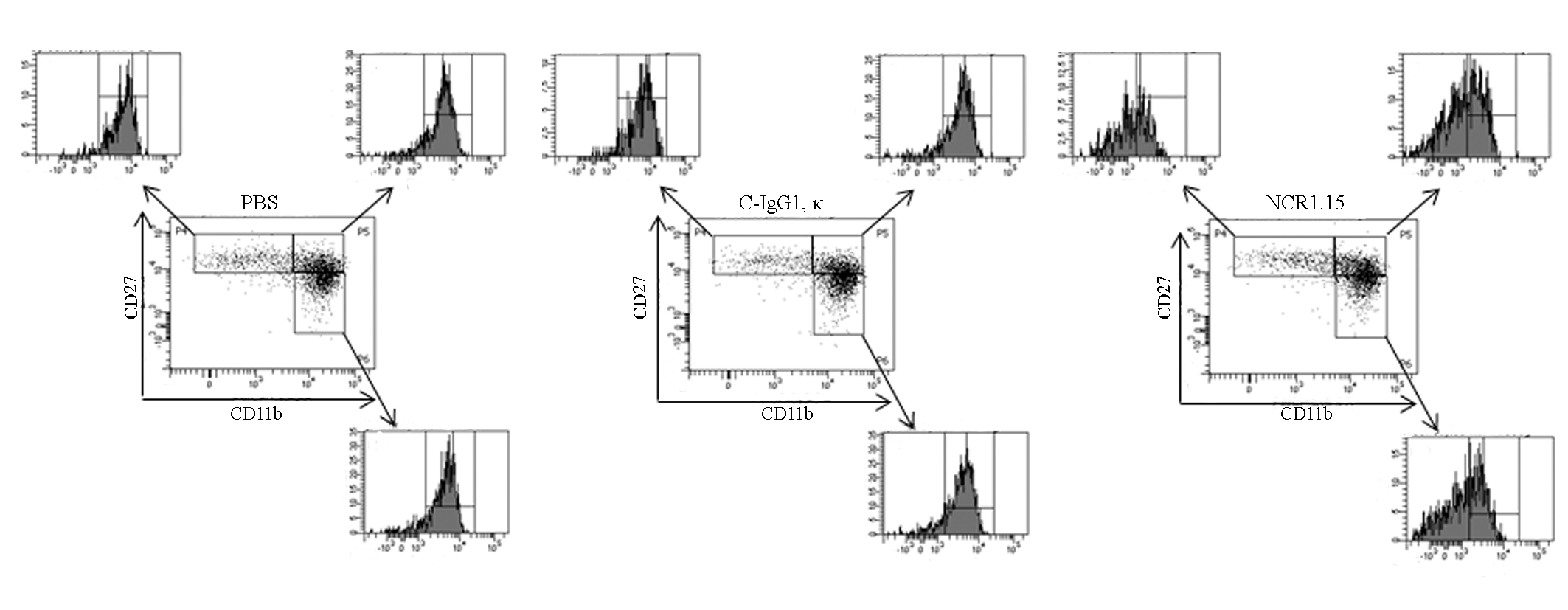

Supplement: S1 Fig — The membrane-associated NKp46 expression was significantly reduced in NCR1.15 treated mice comparing to control treatments in all studied NK developmental stages. (TIF) [file pone.0118936.s001.tif]
